# Simultaneous characterization of porous and non-porous electrodes in microbial electrochemical systems

**DOI:** 10.1016/j.mex.2020.101021

**Published:** 2020-08-05

**Authors:** A. Prado, R. Berenguer, A. Berná, A. Esteve-Núñez

**Affiliations:** a Universidad de Alcalá, Alcalá de Henares, Spain; b IMDEA Agua, Parque Tecnológico de la Universidad de Alcalá, 28805 Alcalá de Henares, Spain; c Instituto Universitario de Materiales, Departamento de Química Física, Universidad de Alicante (UA), Apartado 99, 03080 Alicante, Spain

**Keywords:** Electrode materials, Electroactive biofilms, Bioelectrochemical systems, Porous and non-porous electrodes

## Abstract

*Adequate electrochemical characterization of electrode material/biofilms is crucial for a comprehensive understanding and comparative performance of bioelectrochemical systems (BES). However, their responses are greatly affected by the metabolic activity and growth of these living entities and/or the interference of electrode wiring that can act as an electroactive surface for growth or constitute a source of contamination by corrosion. This restricts the meaningful comparison of the performance of distinct electrode materials in BES. This work describes a methodology for simultaneous electrochemical control and measurement of the microbial response on different electrode materials under the same physicochemical and biological conditions. The method is based on the use of a single channel potentiostat and one counter and reference electrodes to simultaneously polarize several electrode materials in a sole bioelectrochemical cell. Furthermore, various strategies to minimize wiring corrosion are proposed. The proposed methodology, then, will enable a more rigorous characterization of microbial electrochemical responses for comparisons purposes.*

• Experimental Set-up allows to polarize several working electrodes at the same time.
• Chronoamperometry can be performed simultaneously with a potentiostat.
• The physicochemical and biological conditions in each working electrode will be exactly the same

## Graphical Abstract

**Figure:**
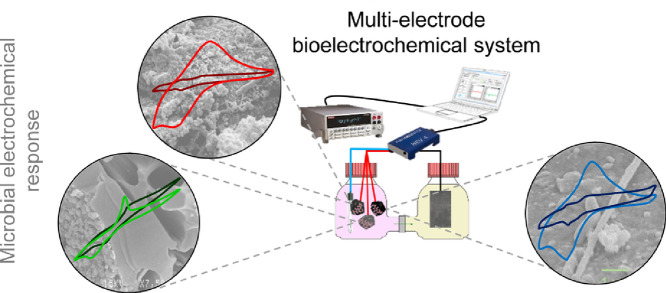

Specifications table

**Table:** 

Subject Area	•Chemical Engineering •Environmental Science •Materials Science
More specific subject area:	Microbial Electrochemical Technologies (METs)
Method name:	A method for multi-electrode polarization and chronoamperometric characterization in microbial electrochemical cells using a single channel potentiostat.
Name and reference of original method	Bard, A. J., Faulkner, L. R.,. Electrochemical methods: fundamentals and applications.2^nd^ Ed., 2001, John Wiley & Sons, New York. Prado, A., Berenguer, R., & Esteve-Núñez, A. (2019). Electroactive biochar outperforms highly conductive carbon materials for biodegrading pollutants by enhancing microbial extracellular electron transfer. Carbon, 146, 597-609.
Resource availability	https://www.sciencedirect.com/science/article/pii/S0008622319301587

## Method details

Electroactive biofilms are of vital importance in the context of fundamental research questions and for their potential exploitation in engineering systems, such as bioelectrochemical systems (BES) [1]. Various electrochemical techniques, like chronoamperometry (CA) and cyclic voltammetry (CV), are powerful tools for the study of extracellular electron transfer (EET) of electroactive bacteria [2]. Direct correlation between the biofilm development and sustained electricity generation along time [3] could be established from analyzing changes in CA and CV response and biofilm coverage for a given electrode. Indeed, the electrode material has a crucial role on the growth of electroactive biofilms and their EET and bioelectricity production capabilities [4].

In order to perform CA and CV analysis for studying the electrode-bacteria interaction, it is required to set-up a three-electrode with: a working electrode (WE), a reference electrode (RE), and a counter electrode (CE). With this set-up, polarization curves can be recorded by using a potentiostat, where 1) the potential difference between WE and RE is controlled, and 2) the electrical current flow between WE and CE is measured [5]. This three-electrode system is called Microbial Electrolytic Cell (MEC).

MECs are constructed using a wide variety of electrode materials and in an ever-increasing diversity of configurations. These systems are operated under several physicochemical conditions that include differences in temperature, pH, final electron acceptor, electrode surface area, reactor size, and operation timescale. In order to compare the results from electrochemical analysis, it is necessary to take into account different parameters such as reference states, internal resistance or power densities derived from polarization curves. Such data have been generally obtained using different experimental conditions, materials and/or configurations, making the interpretation and comparison of results among these systems difficult [6].

The characterization of the microbial electrochemical response of electrode material/biofilms presents various important problems. First, in contrast to inorganic catalytic systems, microbes are living entities and thus may change over time in their composition (variable microbial population) and activity. Therefore, the physiological state, growth phase, and “history” of the microorganisms should be taken into consideration. In addition, planktonic bacteria also play an important role on the bioelectrocatalytic reaction [7]. Hence, it is very difficult to replicate exactly the same biological conditions, like number of bacteria, metabolic activity and growth phase. This fact remarkably hampers the reproducibility of the results of a given MEC and makes it practically impossible to compare the performance of different materials that either have been characterized in different cells or in the same cell but under different operation conditions.

Secondly, a deoxygenated solution is necessary in biological chamber, to keep anaerobic microorganisms viable and to avoid oxygen-related reduction currents/peaks in CVs that could hinder the identification of bioelectrochemical processes. Electrochemical characterization of electrode materials requires them to be immersed in an oxygen-free aqueous electrolyte, so all external connection by using wires or, alternatively, wire-material connectors (like conductive adhesives) are inevitably exposed to water. In other cases, although direct exposure of wire-material junction is avoided, the electrolyte can penetrate through the pores of the material to reach the junction. This contact with the medium usually causes the corrosion of wires and/or connectors, thus, releasing redox active and/or non-biocompatible species that can interfere the bioelectrochemical response of the bacteria. Moreover, corrosion may enhance the electric resistance of these elements, affecting the measurements. Consequently, the recorded electrochemical response for an electrode material/biofilm system might be remarkably affected by corrosion of connecting elements, again hampering the reproducibility and comparability of the electrochemical behavior of materials and systems.

To face these problems, this work presents a new method for the meaningful and comparative characterization of the microbial electrochemical response of different electrode materials [8]. The method is based on the simultaneous electrochemical control and characterization of various distinct material/electroactive bacteria systems in the same reactor, under identical physicochemical and biological conditions, and minimizing wiring-derived corrosion effects.

**Fig. 1:**
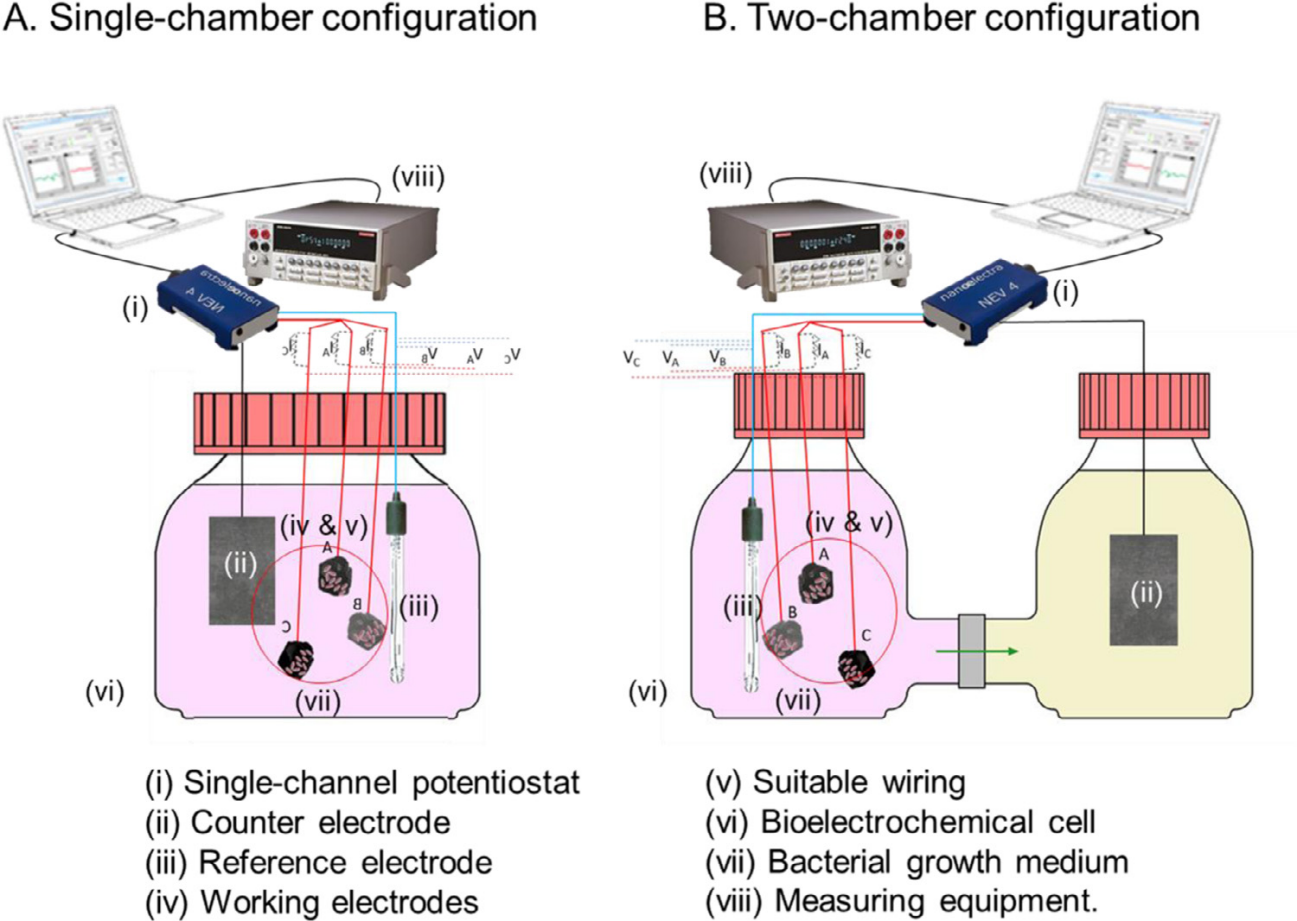
Diagram of the MEC and the connections with the potentiostat and the multimeter.

The method essentially requires the use of a (i) single-channel potentiostat, (ii) one counter electrode and (iii) one reference electrode and (iv) several working electrodes connected by (v) a suitable wiring. All the electrodes are immersed in (vi) a sole bioelectrochemical cell with (vii) bacterial growth in a deoxygenated medium, and their respective electric signals (current, potential, resistance, etc.) are registered by (viii) proper measuring equipment. These elements are schematized in Fig. 1 and operate as a conventional MEC capable to perform classical electroanalytical techniques [5].

(i)-(iv) The single-channel potentiostat is used to impose a fixed or varying potential, enabling to register the overall produced electrical current. Apart from the multiple target working electrodes, a blank electrode showing a well-known characteristic response is also recommended to validate the measurements. The wires connecting each of the working electrode are all merged with the working terminal of the potentiostat. In contrast, the counter and reference electrodes are individually connected to their corresponding pins. During electrochemical measurements, the potentiostat displays the potential difference between the working terminal and the reference electrode. Indeed, the electrical current recorded by the potentiostat adds up all the individual currents passing through each working electrode; hence, such value does not correspond to the real single value for each of the working-reference electrode potential systems.

(v) Wiring the electrode materials entails the creation of conductive, mechanically stable and corrosion-resistant wire-material junctions. The type of wire-material connection will depend on the nature and properties of both the wire and the material, emphasizing their corrosion susceptibility and porosity, respectively. The porosity of the material may play a role, by facilitating the penetration and accessibility of the electrolyte to the wire or junction, and/or by adsorbing and concentrating the released corroded redox-active species on the surface of the electrode. Overall, to avoid corrosion interferences in the presented method, we propose the following two-step strategy for wiring: promotion and subsequent protection of electrical contacts at wire-material junctions. The promotion of electrical contacts can be achieved physically (insert the wire in a drilled hole, tying the wire, etc.), by the aid of conductive adhesives (glues, tapes, etc.), and/or by interposing an additional conductive material which can be better connected to the wire. On the other hand, the protection/isolation of junctions from the electrolyte is generally addressed by coating/sealing with non-corroding resin-like adhesives.

(vi) Different configurations of bioelectrochemical cell can be used. Fig. 1 describes two typical set-ups enabling to work at laboratory scale. The *single-chamber configuration* (Fig. 1A) consists of a unique cell/chamber containing the working electrodes together with the counter and reference electrodes immersed in the same electrolyte. In the *two-chamber configuration* (Fig. 1B), the working and reference electrodes are physically separated from the counter electrode by using two different compartments (the so-called main and auxiliary chambers, respectively) that are ionically interconnected through a suitable polymeric membrane. This last configuration is preferred when the electrochemical processes occurring at the counter electrode can interfere in the characterization of the working electrodes. The cells are usually isolated to keep deoxygenated conditions by hermetic sealing.

Both configurations allow to have all the working electrodes under the same conditions. Physicochemical parameters, such as temperature, pH, solar radiation, presence of gases, concentration of electron donors and salts would be the same, so the bacterial culture inoculum, and its initial metabolic activity, will be the same for all working electrodes. The differences observed in the responses of each of the working electrodes will only be the consequence of the interaction electrode material/bacteria. In this way, possible external factors that may lead to erroneous interpretations are minimized.

(vii) The bacterial growth medium must include the following compounds: salts to assure bacterial viability and to maintain osmotic balance. Furthermore, such salts include pH buffering species. Vitamins and minerals, required in small amounts and necessary for metabolic reactions. Finally, oxidizing and reducing species are necessary. The reducing specie is the carbon source that e microorganisms oxidize. In this particular case, no soluble electron acceptor was added, since the electrode performed this role. This medium must be deoxygenated by flushing N_2_ or Ar, and alternatively, N_2_:CO_2_ in case of using bicarbonate buffer. It is also recommended to keep the medium in continuous agitation with a magnetic stirrer. In this sense, a laminar agitation allows a faster biofilm formation while a turbulent agitation may increase the homogeneity of the medium.

(viii) Measuring equipment, involving various single multimeters or a multichannel multimeter to register the individual currents produced by each of the working electrodes. Some multichannel measuring devices cannot record electrical current signals, and only measuring potential differences is available. In this case, resistors of a well-known value and negligible compared to the whole internal resistance of the system are required, in order to be the measured value for the potential difference corresponding to an electrical current value equal to the real value in absence of that resistor, the so-called shunt resistor. The resistor was placed in series on each working electrode, and potential drop was measured in the shunt resistor. The value of the current produced by each of the working electrodes was calculated using Ohm's Law.

## Method validation

To validate the method, two different measurements were proposed. On the one hand, the prevention of wiring corrosion was validated by using abiotic electrochemical measurements, e.g. cyclic voltammetry. Under these conditions, the absence of microbial electrochemical processes enabled to better discern corrosion-derived currents. On the other hand, the success of using a multielectrode analysis together with a single potentiostat was validated by means of a chronoamperometric experiment in presence of microbial electrochemical processes.

### 1- Strategies for avoiding corrosion in wiring: a CV diagnosis

**Fig. 2:**
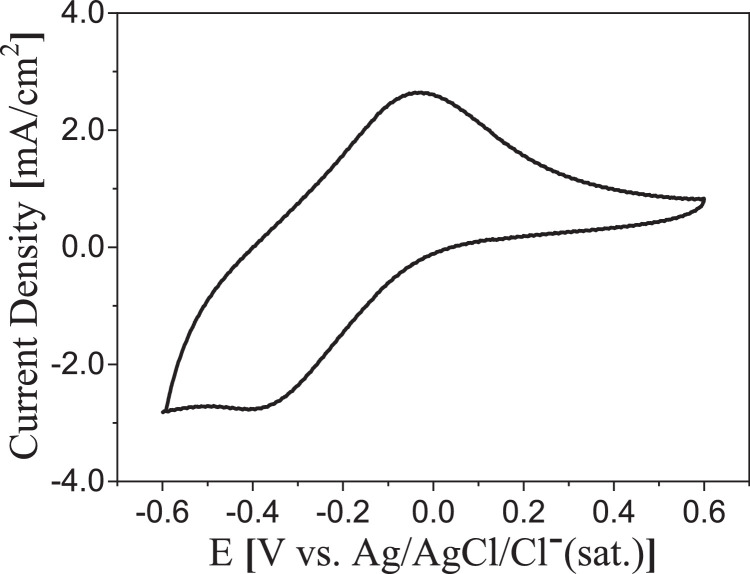
Cyclic voltammogram at 10 mV/s of a graphite electrode after promoted biofilm growth in fresh water medium.

**Fig. 3:**
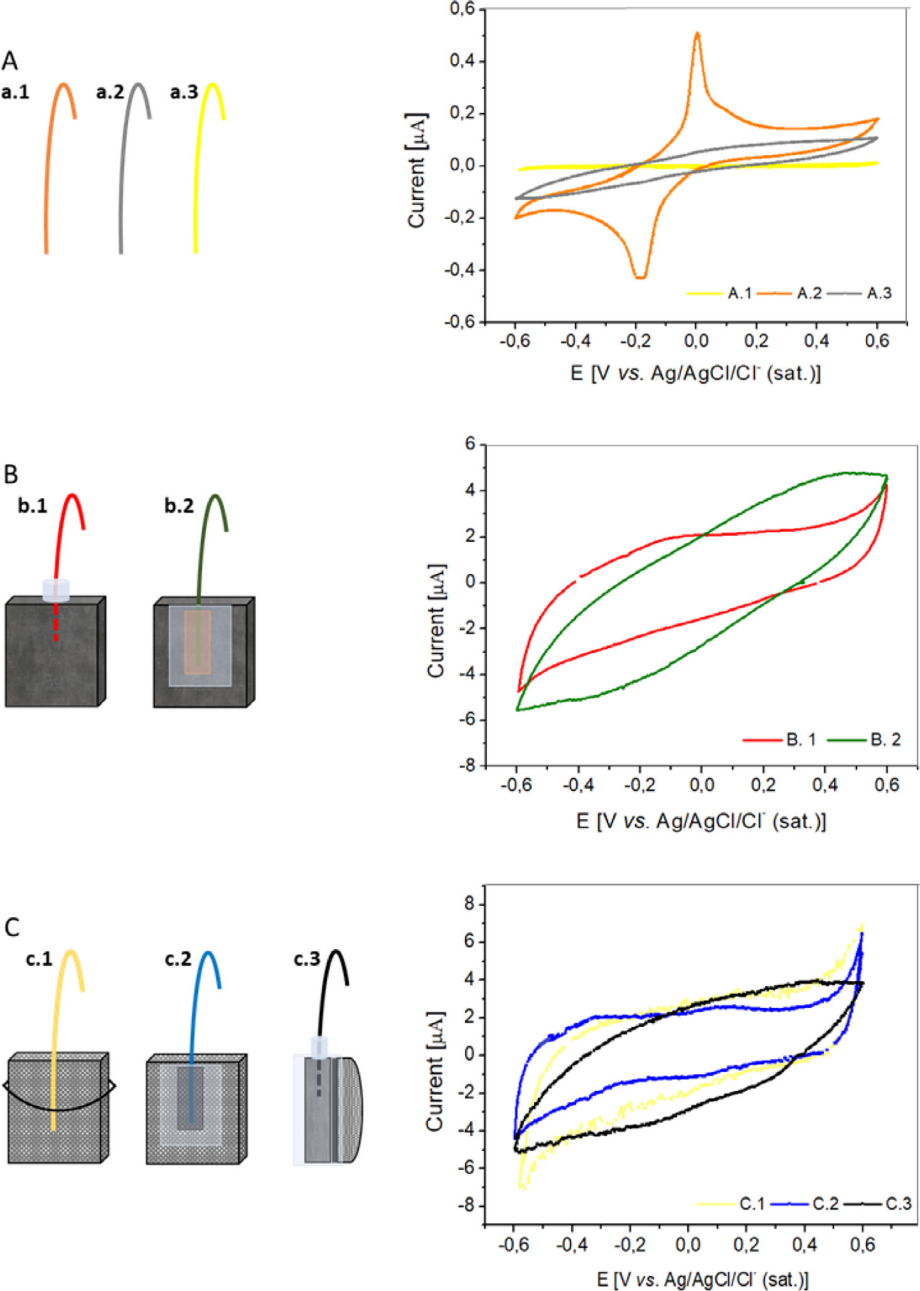
. A) Left: Wires used for working electrode connections: (a.1), copper wire, (a.2) copper wire coated with carbon glue and (a. 3) gold wire. Right: cyclic voltammograms corresponding to left configurations; scan rate = 10 mV s^-1^; 100 mM buffer phosphate. B) Left: Isostatic graphite connected to copper wire and (b.1) sealed with epoxy resin or (b.2) glued with adhesive copper tape and insulated with epoxy resin. Right: cyclic voltammograms corresponding to left configurations; scan rate = 10 mV s^-1^; 100 mM buffer phosphate. C) Left: Porous graphite connected to (c.1) gold wire, (c.2) to copper wire through isolation with carbon glue and epoxy resin, or (c.3) to copper wire through isostatic graphite, carbon glue and epoxy resin. Right: cyclic voltammograms corresponding to left configurations; scan rate = 10 mV s^-1^; 100 mM buffer phosphate.

The corrosion phenomena in the working electrodes is an important phenomenon since a bad wiring would be reporting artefacts, that could be erroneously attributed to the electroactive bacteria activity. This confusion could occur, for example, since the cyclic voltammetry (CV) of a model electroactive bacterium like *Geobacter sulfurreducens* (Fig. 2) [9], presents oxidation and reduction peaks in a potential range very close to those shown occurring with the copper wire CV (Fig. 3A). Therefore, it is important to consider the nature of both the wire and the working electrode material, for deciding how the attachment is made. In this sense, the corrosion of wires and/or wire-material junctions can be easily evidenced by an abiotic voltammetric characterization of the wired electrode materials.

In this context, we validated some strategies to prevent the corrosion of wires and wire-material junctions involving both porous and nonporous materials by using abiotic CV as diagnosis methodology. The CV experiments were carried out in a conventional three-electrodes cell at 10 mV/s in a potential range between -0.6 V and 0.6 V (vs. Ag/AgCl/Cl^−^(sat.)). A HANNA HI-5311 glass body Ag/AgCl/Cl^−^(sat.) electrode with ceramic junction was used as reference electrode, whereas a 2 × 3 cm Ti/Pt mesh, attached to a copper wire protected by heat shrink tubing, acted as a counter electrode. All the electrodes were immersed in phosphate buffer 100 mM deoxygenated with N_2_.

### Wires and connections

To construct the working electrodes, gold and copper were chosen as wire material for their different corrosion susceptibility, while carbonaceous materials were used as electrode materials due to their suitable response in microbial electrochemical systems [8]. We validated the material-wire connection for each electrode according to their voltammetric response (Fig. 3).

A) Wires. The oxidation and reduction processes of the wire material were studied. In the case of gold wire (Fig. 3A-a.3), the CV did not show any peak in the selected potential window demonstrating the absence of oxidation-reduction processes in the potential window explored. In contrast, copper wire showed oxidation-reduction peaks (Fig. 3A-a.1), which could be mistaken as signals provided by electroactive microorganisms. Therefore, this type of wire cannot be used directly with porous carbonaceous materials. To solve this problem, the copper wire should be coated and effectively isolated from the electrolyte with a conductive adhesive, such as PELCOR® Conductive Carbon Glue, avoiding the oxide-reduction processes (Fig. 3A-a.2).

Wire-material connections: In most practical cases the wires are susceptible of corrosion, so the proposed general strategy should be to isolate wires (are exposed at wire-material junctions) from the electrolyte but keeping intact electrical contact. Hence, a wire-material contact must be properly established, physically or assisted by a conductive adhesive, and eventually protected from the electrolyte. The protection is recommended to be extended also to the conductive adhesive in case it might undergo any kind of electrochemical process (interference). However, the type of wire-working electrode connection will depend on the porous or non-porous nature of the carbonaceous material.

B) Connecting wires with non-porous carbonaceous materials. The material used for validation was isostatic Graphite grade 2114-45 provided by Mersen. Due to the non-porous nature of the material, the isolation of connections can be addressed at any point of the electrode surface, independently of whether the junction was on the outer surface or just inserted inside the material. Both two possibilities were tested (Fig. 3B).

• b.1) A 1 mm diameter orifice was drilled in one of the sides of the isostatic graphite plate and a copper wire was inserted for a proper physical contact. Then, the connection was isolated and reinforced on the outer surface of the material in contact with the electrolyte by using Araldit® epoxy.
• b.2) Another possibility was to glue the copper wire to the surface of the isostatic graphite using conductive copper adhesive tape, finally this junction will be covered and isolated with Araldit® epoxy. The response of copper wire-isostatic graphite connections was studied. Our studies revealed that both connections (Fig. 3B) were valid for the construction of working electrodes. Indeed, in spite of the resistance and capacity of the electrode, no oxidation-reduction peaks were observed in the range of selected potentials, so the electrochemical response is truly having a biological origin in the interaction between electroactive microorganisms and electrode surface.

C) Connecting wires with porous carbonaceous materials. The material used for validation was Graphite grade 6506, provided by Mersen. The porous nature of the material enables the electrolyte to permeate through, so the isolation of connections can be exclusively faced just on the junctions (Fig. 3C):

• c.1) In the case of using non-corroding wires, e.g. gold, a simple physical junction could be made to safely avoid any corrosion interference. In contrast, if corroding materials are used as wires, e.g. copper or nickel, the following connections are suggested:
• c.2) the wire should be connected with PELCOR® Conductive Carbon Glue, so the copper wire would be directly attached to the porous carbonaceous material while isolated from the electrolyte. This junction should be coated with Araldit® epoxy to seal and strengthen the connection.
• c.3) An alternative actin would b is to stick the porous carbonaceous material to a current collector (for example, isostatic graphite) with PELCOR® Conductive Carbon Glue, and coat the junction with Araldit® epoxy to isolate and reinforce the connection. Interestingly, the use of appropriated connections revealed the absence of oxidation-reduction peaks and the CV response was pure capacitive (Fig. 3C).

### 2- Method for performing a multielectrode analysis using a single potentiostat

Chronoamperometry is one of the most important characterization techniques of microbial electrochemical systems, so it was used to validate our simultaneous multielectrode analysis. Three different porous carbonaceous materials were used as working electrodes (WE) in a single chamber cell. These materials differed in electrical conductivity (WE1, WE2 and WE3, from lowest to highest electrical conductivity). The wiring was done as described in section one (Fig. 3C-c.2). The counter electrode and reference electrode were identical to those described in section one. The cell had a volume of 500 mL, the electrolyte used was Fresh Water Medium (FWM). FWM contained the following mineral salts (per liter): 2.2 g of Na_2_HPO_4_, 1.18 g of NaH_2_PO_4_, 0.64 g of NH_4_Cl, 0.26 g of KCl and 0.024 g of C_6_H_5_FeO_7_ (ferric citrate). Moreover, it includes a mix of vitamins and trace minerals [10]. Acetate (20 mM) was supplied as the sole carbon and electron donor. Anaerobic conditions were achieved by flushing the culture media with N_2_ to remove oxygen and a phosphate buffer was used to keep a pH of 7. This medium was inoculated with 20 mL of *Geobacter sulfurreducens* pure culture with an OD_600_ = 0.6.

Each working electrode was polarized to +0.2 V (*vs.* Ag/AgCl(sat.)) with a Nanoelectra NEV-4 potentiostat, which allows to monitor the overall electrical current passing through the system. The real potential difference WE-RE and individual current at each working electrode were continuously recorded with a Keithley 2700 multichannel multimeter. The basic model of this family of multimeters has only 2 channels to measure current and 20 channels for recording potential differences. Alternatively, a resistor of known value, shunt resistor can be incorporated to potential-measuring channels for registering the potential drop across it the it is related to the electrical current through Ohm's Law. This resistor is usually called shunt-resistor in electronic field.

Nevertheless, to follow the same measurement strategy (for a better comparability), three resistors were used to measure the current of the three working electrodes. The resistor values should be selected according to the expected currents and the accuracy of the measuring equipment. The resistors were connected in series between each working electrode and in parallel with respect to the multimeter. In addition, another channel of the multimeter was connected in parallel between the working and reference electrodes, to know the real polarization potential for each working electrode. Thus, although the resistance of each material that is acting as working electrode is different, it can be checked if electrodes are polarized at the right potential. Finally, to verify that the current produced by the system is being correctly monitored, one of the multimeter current channels was connected, in series, to the counter electrode. The sum of the current generated by each working electrode should be equal to both the current registered at the counter electrode and the current registered by the potentiostat.

**Fig. 4:**
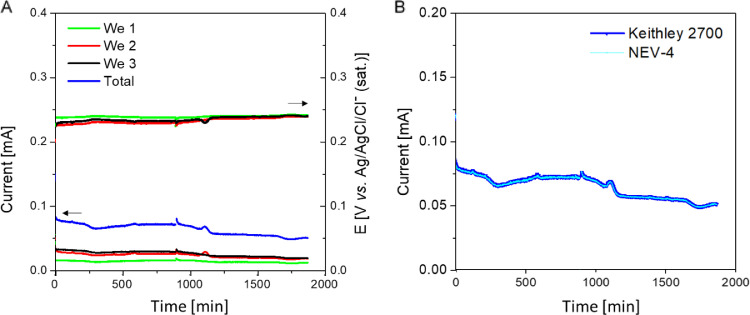
(A) Current intensity and polarization potential associated to the 3 working electrodes of the MEC including the summation of all currents. (B) Comparison of the total current recorded by both the multimeter and the potentiostat.

Fig. 4A shows the calculated values of current produced by each of the three working electrodes, the bigger electrical current value corresponds to the electrode material with lower electrical resistance. These values were calculated through the instantaneous potential difference measured in each working electrode and the value of the resistor used in the external circuit (1 KΩ). The total sum of the currents produced by each working electrode measured by the aid of the Keithley multimeter (blue line in Fig. 4A) was the same as the one recorded by the NEV-4 potentiostat (see fitting in Fig. 4B). Furthermore, the real potential ​​of each working electrode remained constant around 0.23 V over time for the different electrodes (Fig. 4A). The difference of 30 mV between the set and measured potentials is assigned to the inevitable circuit and connections resistances, but this small value supports that the electrochemical characterization based on the proposed method has been carried out successfully.

## Declaration of Competing Interest

None
